# Revealed preferences towards the appraisal of orphan drugs in Poland - multi criteria decision analysis

**DOI:** 10.1186/s13023-018-0803-9

**Published:** 2018-04-27

**Authors:** Katarzyna Kolasa, Krzysztof Miroslaw Zwolinski, Vladimir Zah, Zoltán Kaló, Tadeusz Lewandowski

**Affiliations:** 1 Department of Health Economics and Healthcare Management, Kozminski University, Warsaw, Poland; 2 Advanced Management Training Programme in Pharmacoeconomics, Pharmaceutical Marketing and Law, Warsaw University of Technology, Warsaw, Poland; 3 HEOR, ZRx Outcomes Research, Toronto, Canada; 4 Department of Health Policy & Health Economics, Faculty of Social Sciences, Eötvös Loránd University, Budapest, Hungary; 5 Department of Econometrics, Poznan University of Economics, Poznan, Poland; 6 Syreon Research Institute, Poznan University of Economics, Budapest, Hungary

**Keywords:** Revealed preferences, Multi criteria decision making, Orphan drugs, Health technology assessment, Poland, Economic and non-economic decision making criteria

## Abstract

**Background:**

A Multi Criteria Decision Analysis (MCDA) technique was adopted to reveal the preferences of the Appraisal Body of the Polish HTA agency towards orphan drugs (OMPs).

**Results:**

There were 34 positive and 23 negative HTA recommendations out of 54 distinctive drug-indication pairs. The MCDA matrix consisted of 13 criteria, seven of which made the most impact on the HTA process. Appraisal of clinical evidence, cost of therapy, and safety considerations were the main contributors to the HTA guidance, whilst advancement of technology and manufacturing costs made the least impact.

**Conclusions:**

MCDA can be regarded as a valuable tool for revealing decision makers’ preferences in the healthcare sector. Given that only roughly half of all criteria included in the MCDA matrix were deemed to make an impact on the HTA process, there is certainly some room for improvement with respect to the adaptation of a new approach towards the value assessment of OMPs in Poland.

**Electronic supplementary material:**

The online version of this article (10.1186/s13023-018-0803-9) contains supplementary material, which is available to authorized users.

## Background

There are up to 36 million individuals suffering from as many as 5000–8000 different types of rare diseases in the EU. Although many of them significantly impact life expectancy, available data suggests that there is still considerable number of patients that remains without the treatment [1].

Given the growing challenges with limited budgets being spent on innovative orphan medicine products (OMPs), more transparent and objective pricing & reimbursement (P&R) decision-making process is sought. Among new initiatives, the Transparent Value Framework (TVF) developed by the Working Group on Mechanism for Coordinated Access to OMPs (MoCA) could be pointed out [2]. The TVF directs special attention towards the inclusion of non-economic criteria, such as unmet medical needs and availability of alternative treatment options in the P&R process of OMPs. It attempts to evaluate feasibility of the inclusion of a broad scope of decision-making criteria in the efforts towards more transparent and equitable assessment of the value of OMPs.

Despite limited number of examples of specific P&R frameworks being established for the evaluation of OMPs, majority of jurisdictions have not separated reimbursement processes for OMPs and non-OMPs [3–5]. With that, it is interesting to ask what kind of criteria is utilized for the assessment of OMPs in the P&R system without any special arrangements for the evaluation of treatments for rare diseases. It is particularly valuable to provide a relevant insight into whether the broader set of criteria as envisaged by the MoCA project is at least implicitly taken into consideration in the appraisal processes of OMPs.

Central and Eastern European (CEE) settings can serve as an interesting research field for that purpose. On the one hand, criteria of clinical and cost effectiveness analysis alongside budget impact analysis are commonly established in the format of standard HTA, and there are some instances where the fixed cost effectiveness threshold is explicitly set amongst P&R rules for both orphan and non-orphan drug technologies [6, 7]. On the other hand, CEE countries lag behind the rest of the EU with regard to accessibility to treatment for patients with rare diseases. For instance, it was revealed that only in 22 out of registered 61, and 28 out of registered 72 OMPs were available in Bulgaria and Latvia respectively [8–10]. According to the EURORDIS published study, as many as 90% of 60 OMPs were covered by public funds in the Netherlands, whilst only 30% were covered in Romania [10].

The Polish jurisdiction was chosen for the purpose of this study. It is the biggest country in the CEE Region with a well-established HTA process. The proceedings from the P&R negotiations are not available in the public domain. In contrast to other countries in the CEE Region, the Polish HTA Agency does, however, publish its recommendations.

The objective of our study was to reveal the value criteria appraised in the HTA appraisal process in Poland. The Polish Appraisal Body consists of the President of the HTA agency and ten members of the Appraisal Committee. Among them, there are representatives of the Ministry of Health, the National Health Fund, regulatory body, and patients’ ombudsman [11]. The HTA recommendations are not mandatory in Poland. The pricing negotiations are conducted at the discretion of the Ministry of Health.

The Multi Criteria Decision Analysis (MCDA) framework was chosen for the purpose of the study. There is an increasing interest in the MCDA’s implementation into the P&R process as it allows for a trade-off between different decision-making criteria [12]. In the particular case of our research, it provides an opportunity to reveal the preferences towards value criteria, and to assign weights to each of them representing their importance in the HTA appraisal process. It was hypothesized that findings from our research can help elicit recommendations on how to achieve more transparency in the pricing and reimbursement process for OMPs.

## Methods

The analysis was divided into five steps following the ISPOR checklist designed for MCDA studies [15]:

1. Description of the decision problem,
2. Selecting and defining criteria,
3. Measuring performance,
4. Scoring and weighting criteria,
5. Dealing with uncertainty.

The MCDA analysis was performed utilizing validated ZRx MCDA tool [13].

### Description of the decision problem

The decision problem was set to reveal the OMPs’ value criteria appraised in the HTA process between January 2008 and June 2015 by the Polish HTA Agency (AHTAPoL).

#### MCDA matrix development

An MCDA matrix was conceived based on the set of value attributes of the OMPs identified in the systematic review of the literature published elsewhere [14]. The pilot review of 10 HTA recommendations was conducted to reveal potential additional criteria utilized in the appraisal process. Following ISPOR’s guidelines, the attributes were chosen with respect to certain principles such as completeness, non-redundancy, non-overlap, and preferential independence [15].

### Measuring performance

#### Review of HTA recommendations

A database of HTA recommendations issued with respect to OMPs was established. Each indication for the same active substance was considered separately and treated as a distinctive case (drug-indication pair). HTA recommendations issued by both the Appraisal Body and, if available, by the President of the Polish HTA agency, were used for the purpose of the study (HTA outcomes). The categorization of HTA outcomes into negative and positive ones was carried out, and reasons for each recommendation were elicited [16].

#### MCDA matrix population

Two independent reviewers conducted the review of HTA recommendations to identify which MCDA criteria were considered in the HTA process for each drug-indication pair separately. Any disagreement was resolved by consensus. An MCDA matrix was established in the table format known as “matrix performance” [17]. It was populated with the data from each HTA recommendations so that one of three different numbers could be assigned to each drug-indication pair for each MCDA criteria separately: 1- full deliberation, in case a given criteria was utilized to justify the HTA recommendation, 0.5- partial deliberation, in case a given criteria was only mentioned in the HTA report without being used for justification of the HTA recommendation, and 0- lack of deliberation.

### Scoring and weighting criteria

#### MCDA analysis

Multiple Attribute Value Theory (MAVT) was adopted to reveal the preferences of the HTA Appraisal Body. This is a methodology designed to address complex decision making problems involving multiple attributes. In principle, it compares different alternatives against each other using a number of distinctive criterions. A simple linear additive model (SLAM) and analytic hierarchy process (AHP) were adopted for the purpose of this study [18]. The SLAM method evaluates alternatives against a chosen set of criteria taking into consideration both preferences for each criteria (weights) and performance of each alternative against those criteria. We utilized SLAM method to compare the frequency of deliberation of each criterion (model’s alternatives) in HTA process across all studied cases (alternatives’ performance). It was assumed that each drug indication pair contributed equally to the analysis (model’s weights); hence, equal weights were assigned to each HTA recommendation. In contrast to SLAM, AHP analysis is based on a special matrix that allows measuring the intensity of importance of each MCDA criteria in a pair-wise comparison. In that study, the relative importance of each criterion against another was ranked on the scale that corresponds to the number of drug indications pairs used.

A normalized matrix allowed assigning weight to each criteriion based on its importance in the HTA appraisal process as well. The sum of the weights equalled 100%. For both SLAM and AHP, the frequency of the deliberation of each MCDA criterion between groups of positive and negative HTA outcomes was compared. In cases of a lack of significant differences, an MCDA analysis was conducted jointly for both groups. Otherwise, a separate MCDA processes was planned. Further methodological details regarding MCDA analysis applied in the study are presented in the Additional file 1.

### Dealing with uncertainty

#### Sensitivity analysis

In the sensitivity analysis, both the Variable Interdependent Parameters (VIP) and maximal regret methods were utilized. For the purpose of this particular study, we assumed a threshold of 0.5 for both VIP minimum value and maximal regret to assess the importance of the given criteria. Further details are available in the supplementary materials.

## Results

### Data

The MCDA matrix consisted of 13 criteria, ten of which were imported from the other study [14]. Additionally, three criteria were added as a result of the review of ten randomly selected HTA recommendations. It was therapy costs, the impact of other HTA guidelines on the Polish HTA appraisal, and the results of rationalization analysis whose objective is to identify financial sourcing for the technology in question. The definition of the remaining ten criteria is presented elsewhere [14].

In total, 57 distinctive drug-indication pairs were included in the dataset. Based on the review of HTA recommendations, 34 positive and 23 negative HTA outcomes were distinguished (Table 1).

**Table 1:** HTA outcomes for OMPs issued by the Polish HTA agency between

Entry	Brand name (Active substance)	Indication	HTA outcome	Data source	Reason for HTA recommendation
1	Adcetris (brentuximab vedotin)	Lymphoma CD30+: Hodgkin Disease (C81), Lymphoma, Non-Hodgkin (C84.5)	Negative	Rekomendacja prezesa AOTM nr 96/2013	Clinical reasons: insufficient evidence for use (poor quality data). Economic reasons: insufficient justification of the treatments cost in relation to its benefit
2	Adempas (riociguat)	Chronic Thromboembolic Pulmonary Hypertension (CTEPH) (ICD-10 I27, I27.O and/or I26)	Positive	Rekomendacja prezesa AOTM nr 261/2014	Minor restrictions: use at lower price
3	Arzerra (ofatumumab)	Chronic Lymphocytic Leukemia in patients who are refractory to fludarabine and alemtuzumab	Negative	Rekomendacja prezesa AOTM nr 5/2012	Clinical reasons: insufficient evidence for use (poor quality data). Economic reasons: unacceptable budget impact, insufficient justification of the treatments cost in relation to its benefit
4	Atriance (Nelarabine)	treatment of patients with T-cell acute lymphoblastic leukaemia (T-ALL) and T-cell lymphoblastic lymphoma (T-LBL) whose disease has not responded to or has relapsed following treatment with at least two chemotherapy regimens, eligible for a bone marrow transplant	Positive	Stanowisko Rady Konsultacyjnej nr 13/04/2009	Minor restrictions: use at lower price
5	Bramitob (tobramycin)	treatment of Pseudomonas aeruginosa lung infection in cystic fibrosis (ICD-10 E84)	Negative	Rekomendacja prezesa AOTM nr 83/2013	Economic reasons: insufficient justification of the treatments cost in relation to its benefit
6	Cometriq (cabozantinib)	Thyroid Neoplasms (ICD-10 C73)	Negative	Rekomendacja prezesa AOTM nr 51/2015	Clinical reasons: insufficient evidence for use (inappropriate comparator or poor quality data), poor safety. Economic reasons: poor economic data, insufficient justification of the treatments cost in relation to its benefit
7	Cystadane (Betaine anhydrous)	Homocystinuria	Positive	Rekomendacja prezesa AOTM nr 6/2010, Stanowisko Rady Konsultacyjnej nr 9/3/2010	Minor restriction: monitoring required
8	Elaprase (Idursulfase)	Mucopolysaccharidosis type II, MPS II (Hunter syndrome) – long-term treatment	Negative	Komunikat na stronie www AOTM	Clinical reasons: insufficient evidence for use (poor quality data)
9	Esbriet (pirfenidone)	Idiopathic Pulmonary Fibrosis (ICD-10 J 84.1)	Negative	Rekomendacja prezesa AOTM nr 79/2013	Clinical reasons: insufficient evidence for use (poor quality data), poor safety
10	Evoltra (clofarabine)	Treatment of acute lymphoblastic leukaemia (ALL) in paediatric patients who have relapsed or are refractory after receiving at least two prior regimens and where there is no other treatment option anticipated to result in a durable response, in patients eligible for a hemapoietic stem cell transplant	Positive	Rekomendacja prezesa AOTM nr 127/2012	Major restriction: used restricted to specific subpopulation
11	Exjade (deferasirox)	Treatment of chronic iron overload	Positive	Rekomendacja prezesa AOTM nr 68/2012	Minor restriction: monitoring required
12	Fabrazyme (Agalsidase beta)	Fabry disease (alpha-galactosidase A deficiency) – long-term replace therapy	Negative	Stanowisko Rady Konsultacyjnej nr 20/06/2009	Clinical reasons insufficient evidence for use (inappropriate comparator or poor quality data), poor safety. Economic reasons: insufficient justification of the treatments cost in relation to its benefit
13	Firazyr (icatibant)	Treatment of acute attacks of hereditary angioedema (HAE) in adults (with C1-esterase-inhibitor deficiency)	Negative	Rekomendacja prezesa AOTM nr 22/2015	Economic reasons: insufficient justification of the treatments cost in relation to its benefit
14	Gazyvaro (obinutuzumab)	Chronic lymphocytic leukaemia (CLL) (ICD-10: C.91.1)	Negative	Rekomendacja prezesa AOTM nr 60/2015	Clinical reasons: insufficient evidence for use (inappropriate comparator or poor quality data), poor safety. Economic reasons: poor economic data, insufficient justification of the treatments cost in relation to its benefit
15	Glivec (Imatinib)	Myelodysplastic/myeloproliferative diseases (MDS/MPD)	Positive	Rekomendacja prezesa AOTM nr 7/2011	Unrestricted
16	Glivec (Imatinib)	Dermatofibrosarcoma protuberans (DFSP)	Positive	Rekomendacja prezesa AOTM nr 5/2011	Unrestricted
17	Glivec (Imatinib)	Malignant gastrointestinal stromal tumors (GIST)	Positive	Komunikat wraz z uzasadnieniem na stronie AHTAPol	Unrestricted
18	Glivec (Imatinib)	Philadelphia chromosome positive chronic myeloid leukemia (ALL Ph+)	Positive	Rekomendacja prezesa AOTM nr 6/2011	Unrestricted
19	Increlex (Mecasermin)	Insulin-like growth factor deficiency –IGF-1 (Laron Syndrome) – long-term treatment	Positive	Stanowisko Rady Konsultacyjnej nr 43/12/2009	Major restriction: resubmission required after certain time
20	Jakavi (ruxolitinib)	primary myelofibrosis (also known as chronic idiopathic myelofibrosis), post-polycythaemia-vera myelofibrosis or post-essential-thrombocythaemia myelofibrosis	Positive	Rekomendacja prezesa AOTM nr 120/2014	Unrestricted
21	Kalydeco (ivacaftor)	Cystic fibrosis (CF) (ICD-10 E84)	Negative	Rekomendacja prezesa AOTM nr 54/2015	Clinical reasons: insufficient evidence for use (poor quality data). Economic reasons: insufficient justification of the treatments cost in relation to its benefit
22	Kuvan (sapropterin)	Hyperphenylalaninemia (HPA) in patients with tetrahydrobiopterin (BH4) deficiency	Positive	Rekomendacja prezesa AOTM nr 55/2011	Major restriction: resubmission required after certain time
23	Mabthera (rituximab)	Non-Hodgkin’s lymphoma (NHL)	Positive	Rekomendacja prezesa AOTM nr 7/2012	Major restriction: used restricted to specific subpopulation
24	Mabthera (rituximab)	Non-Hodgkin’s lymphoma classified to code ICD-10 C84	Negative	Rekomendacja prezesa AOTM nr 24/2012	Clinical reason: insufficient evidence for use (inappropriate comparator or poor quality data)
25	Mabthera (rituximab)	Code ICD-10 C85 (Other and unspecified types of non-Hodgkin lymphoma)	Positive	Rekomendacja prezesa AOTM nr 25/2012	Unrestricted
26	Mabthera (rituximab)	Hodgkin Lymphoma (Hodgkin disease-HD)	Positive	Rekomendacja prezesa AOTM nr 19/2012	Major restriction: used restricted to specific subpopulation
27	Mepact (mifamurtide)	Osteosarcoma (ICD-10 C40–41)	Negative	Rekomendacja prezesa AOTM nr 78/2013	Clinical reasons: insufficient evidence for use (poor quality data). Economic reasons: insufficient justification of the treatments cost in relation to its benefit
28	Mozobil (plerixafor)	In combination with granulocyte-colony-stimulating factor to enhance mobilisation of haematopoietic stem cells to the peripheral blood for collection and subsequent autologous transplantation in patients with lymphoma and multiple myeloma whose cells mobilise poorly (ICD-10: C81–85, C90)	Negative	Rekomendacja prezesa AOTM nr 182/2013	Clinical reason: poor safety. Economic reasons: insufficient justification of the treatments cost in relation to its benefit
29	Myozyme (alglucosidase alfa)	Pompe disease (acid-α-glucosidase deficiency) (ICD-10 E74.0)	Negative	Rekomendacja prezesa AOTM nr 8/2013	Clinical reason: insufficient evidence for use (poor quality data). Economic reasons: insufficient justification of the treatments cost in relation to its benefit
30/31	Nexavar (Sorafenib)	Renal cell carcinoma (RCC)	Negative	Rekomendacja prezesa AOTM nr 48/2009, Stanowisko Rady Konsultacyjnej nr 27/10/26/2009, and Uchwała Rady Konsultacyjnej nr 22/07/2008	Clinical reasons: insufficient evidence for use (poor quality data), poor efficacy. Economic reasons: insufficient justification of the treatments cost in relation to its benefit
32	Nexavar (Sorafenib)	Hepatocellular carcinoma (HCC)	Positive	Rekomendacja prezesa AOTM nr 26/2010	Major restriction: used restricted to specific subpopulation
33	Nplate (Romiplostim)	Chronic immune (idiopathic) thrombocytopenic purpura (ITP)	Positive	Rekomendacja prezesa AOTM nr 13/2010	Major restriction: used restricted to specific subpopulation
34	Opsumit (macitentan)	long-term treatment of pulmonary arterial hypertension (PAH) in combination (ICD-10 I27, I27.0)	Negative	Rekomendacja prezesa AOTM nr 23/2015	Clinical reasons: insufficient evidence for use (inappropriate comparator or poor quality data). Economic reasons: unacceptable budget impact
35	Revatio (Sildenafil)	Pulmonary arterial hypertension (PAH)	Positive	Uchwała Rady Konsultacyjnej nr 1/01/2008	Unrestricted
36	Revlimid (Lenalidomide)	Myelodysplastic/Myeloproliferative syndrome (MM/S) (off-label indication)	Positive	Rekomendacja prezesa AOTM nr 83/2011	Off-label indication. Major restriction: used restricted to specific subpopulation. Minor restriction: use at lower price
37	Revlimid (Lenalidomide)	Myelodysplastic/Myeloproliferative syndrome (MM/S)	Positive	Rekomendacja prezesa AOTM nr 11/2012	Major restriction: used restricted to specific subpopulation. Minor restriction: use at lower price
38	Signifor (pasireotide)	Cushing’s disease for whom surgery is not an option or for whom surgery has failed (ICD-10 E 24.0)	Positive	Rekomendacja prezesa AOTM nr 99/2013	Minor restriction: use at lower price
39	Somavert (Pegvisomant)	Acromegaly	Negative	Rekomendacja prezesa AOTM nr 4/2011	Clinical reasons: insufficient evidence for use (inappropriate comparator or poor quality data), poor efficacy. Economic reasons: insufficient justification of the treatments cost in relation to its benefit
40	Sprycel (Dasatinib)	Chronic myeloid leukemia (CML)	Positive	Uchwała Rady Konsultacyjnej nr 23/07/2008	Major restriction: use only as second or subsequent line treatment
41	Sprycel (dasatinib)	Indication clacisified to codes: ICD10:C96.2, within non-standard chemiotherapy programme	Negative	Rekomendacja prezesa AOTM nr 66/2014	Clinical reasons: poor efficacy
42	Sutent (sunitinib)	unresectable or metastatic malignant gastrointestinal stromal tumors (GIST) in adults with disease progression	Positive	Rekomendacja prezesa AOTM nr 20/2012	Unrestricted
43	Tasigna (Nilotinib)	Chronic myeloid leukemia (CML) with resistance or intolerance to prior therapy	Positive	Uchwała Rady Konsultacyjnej nr 53/15/2008	Major restriction: use only if intolerant to other treatment. Minor restriction: use at lower price
44	Thalidomide Celgene (thalidomide)	In combination with melphalan and prednisone as first-line treatment of patients with untreated multiple myeloma aged ≥65 years or ineligible for high-dose chemotherapy	Positive	Rekomendacja prezesa AOTM nr 106/2012	Minor restriction: use at lower price
45	Torisel (Temsirolimus)	Renal cell carcinoma (RCC)	Negative	Rekomendacja prezesa AOTM nr 47/2009. Stanowisko Rady Konsultacyjnej nr 26/10/26/2009	Clinical reasons: insufficient evidence for use (inappropriate comparator or poor quality data). Economic reasons: insufficient justification of the treatments cost in relation to its benefit
46	Torisel (temsirolimus)	treatment of adult patients with advanced renal-cell carcinoma (RCC) with unfavorable prognostic (ICD-10: C64) (RCC-up)	Negative	Rekomendacja prezesa AOTM nr 58/2013	Clinical reasons: insufficient evidence for use (inappropriate comparator or poor quality data), poor safety. Economic reasons: insufficient justification of the treatments cost in relation to its benefit
47	Tracleer (Bosentan)	Pulmonary arterial hypertension (PAH)	Positive	Uchwała Rady Konsultacyjnej nr 1/01/2008	Major restrictions: used restricted to specific subpopulation, use only as second or subsequent line treatment. Minor restriction: monitoring required.
48	Trisenox (arsenic trioxide)	for induction of remission and consolidation in adult patients with pro-Myelotic Leucaemia (APL)/ Retinoic-Acid receptor-alpha PML/RAR alpha	Negative	Rekomendacja prezesa AOTM nr 6/2012	Clinical reason: insufficient evidence for use (poor quality data). Economic reason: insufficient justification of the treatments cost in relation to its benefit
49	Ventavis (Iloprost)	Pulmonary arterial hypertension (PAH)	Positive	Uchwała Rady Konsultacyjnej nr 1/01/2008	Major restrictions: used restricted to specific subpopulation, use only as second or subsequent line treatment. Minor restriction: monitoring required.
50	Vidaza (Azacitidine)	Acute myelogenous leukemia (AML)	Positive	Rekomendacja prezesa AOTM nr 18/2011	Unrestricted
51	Volibris (Ambrisentan)	Pulmonary arterial hypertension (PAH) (ICD-10 I27, I27.0)	Positive	Rekomendacja prezesa AOTM nr 29/2010	Unrestricted
52	Votubia (everolimus)	Treatment of patients with subependymal giant cell astrocytoma (SEGA) associated with tuberous sclerosis complex (TSC) who require therapeutic intervention but are not amenable to surgery (ICD-10 Q85.1)	Positive	Rekomendacja prezesa AOTM nr 81/2014	Minor restriction: use at lower price
53	Vpriv (velaglucerase alfa)	Gaucher disease (ICD-10: E-75)	Positive	Rekomendacja prezesa AOTM nr 120/2013	Minor restriction: use at lower price
54	Yondelis (Trabectedin)	Soft tissue sarcoma	Positive	Rekomendacja prezesa AOTM nr 19/2011	Major restriction: used restricted to specific subpopulation. Minor restriction: use at lower price
55	Zavesca (Miglustat)	Niemann-Pick type C syndrome (disease)	Positive	Rekomendacja prezesa AOTM nr 20/2011	Major restriction: resubmission required after certain time Minor restrictions: use at lower price, monitoring required
56	Xagrid (anagrelide)	Indication classified to codes: ICD-10: D.45 with extensions and D.47 with extensions	Positive	Rekomendacja prezesa AOTM nr 142/2013	Unrestricted
57	Xagrid (anagrelide)	Chronic myeloid leukemia (CML) (ICD-10 C92.1)	Negative	Rekomendacja prezesa AOTM nr 161/2013	Clinical reasons: insufficient evidence for use (poor quality data)

In 19 out of 23 negative HTA outcomes, insufficient clinical evidence was discussed (Table 1). Economic concerns were mentioned on seven occasions, and that was the sole reason for rejection in three cases. The unfavorable results of a cost effectiveness analysis were mentioned most frequently amongst the non-clinical considerations.

In 15 out of 34 positive HTA outcomes, no restrictions were mentioned (Table 1). Among the remaining ones, the recommendation for use limited to certain subgroups of patients was the most often raised suggestion. The Appraisal Body required lowering the cost of therapy in nine cases.

### MCDA analysis

The deliberation of MCDA criteria between drug indication pairs with positive and negative HTA outcomes was compared. Apart from the consideration of safety aspects and the results of rationalization analysis, no significant differences were observed between both groups (Table 2). Consequently, MCDA analysis was conducted jointly for the total dataset.

**Table 2:** MCDA criteria, Test Anova, SLAM, AHP weights and the results of sensitivity analysis

	Test Anova	SLAM	AHP weights	VIP min	VIP max	Max Regret
Indication uniquenes	0.8529	10	2.31%	0.12	0.5	0.87
Disease rarity	0.4815	8	3.34%	0.3	0.66	0.69
Disease severity	0.4837	9	3.38%	0.23	0.5	0.76
Adv.tech.	0.7574	12	0.42%	0	0.05	1.00
Manufacturing technology	0.2169	13	0.23%	0	0.02	1.00
Therapeutic alternative	0.3666	4	8.56%	0.5	0.67	0.5
Sci. evid. Clin.eff	0.2546	1	14.76%	0.98	1.00	0.00
Benefits from use of medicine (safety aspects)	0.0071*	3	12.68%	0.5	0.81	0.5
Cost effectiveness analysis	0.0747	5	9.86%	0.48	1.00	0.52
Budget impact analysis	0.646	6	10.26%	0.44	0.58	0.56
Therapy cost	0.2937	2	17.60%	0.5	0.82	0.5
HTA recommendations issued elsewhere	0.0691	7	11.88%	0.48	0.83	0.51
Rationalization analysis	0.0176*	11	4.73%	0.13	0.5	0.87

*SLAM* Simple Linear Additive Model, *AHP* Analytic Hierarchy Process, *VIP* Variable Interdependent Parameters, *Sci. evid. clin.eff* Scientific evidence for clinical efficiency, *Adv.tech.* Advancement of technology

*statistical significance at *p*-value (≤ 0.05)

The results of the SLAM analysis are presented in Table 2. They indicate that clinical evidence was the most frequently deliberated criteria in the Polish HTA Appraisal process (Table 2). This was followed by the notion of therapeutic costs, consideration of safety aspects, and the availability of alternative treatment options. The data regarding manufacturing technology, advancement in technology, and the results of the rationalization analysis were deemed to be the least important.

The AHP results are presented in Table 3. The matrix of pairwise comparison revealed that the value of the clinical evidence outweighed all other attributes in the HTA process. Its importance ranged from being 1.3 to 55 times higher than safety and manufacturing technology aspects respectively. With a weight of 17%, clinical evidence had the greatest impact on the outcome of the HTA process (Table 4), whilst therapeutic costs and safety aspects, with weights of 14% and 13% respectively, contributed significantly to the HTA outcome as well (Table 4). The pairwise comparison revealed that both criteria were less important than clinical evidence but more important than all the remaining ones. The significance of their value against other attributes ranged from 1.5 to 46 times compared to CEA results and manufacturing costs respectively. With a weight of 10%, availability of therapeutic alternative was the fourth most impactful contributor to the HTA recommendation process (Table 4). Although it was less important than clinical evidence, safety, and therapeutic costs, according to the pairwise comparison, it was still more influential than nine others. The importance of therapeutic alternative was valued at almost twice as much as the one for CEA and BIA, and more than 30 times greater than the manufacturing technology (Table 3). As far as CEA and BIA results are concerned, they almost equally contributed to the HTA recommendation process with a weight of 9% each (Table 4). Although they informed the HTA process to a lesser extent than the four above discussed criteria, both CEA and BIA results were more important than other attributes in half of the cases of pairwise comparisons (Table 3). The advancement in technology and manufacturing technology were found to be amongst the least impactful factors (Tables 3 and 4).

**Table 3:** AHP results - pairwise comparison

MCDA criteria	Indication uniqueness	Disease rarity	Disease severity	Adv.tech.	Manufacturing technology	Therapeutic alternative	Sci. evid. Clin.eff	Benefits from use of medicine (safety aspects)	Cost effectiveness analysis	Budget impact analysis	Therapy cost	HTA recommendations issued elsewhere	Rationali-zation analysis
Indication uniqueness	1	0.763157895	0.828571429	7.25	14.5	0.420289855	0.263636364	0.333333333	0.467741935	0.491525424	0.315217391	0.517857143	1.45
Disease rarity	1.310344828	1	1.085714286	9.5	19	0.550724638	0.345454545	0.436781609	0.612903226	0.644067797	0.413043478	0.678571429	1.9
Disease severity	1.206896552	0.921052632	1	8.75	17.5	0.507246377	0.318181818	0.402298851	0.564516129	0.593220339	0.380434783	0.625	1.75
Adv.tech.	0.137931034	0.105263158	0.114285714	1	2	0.057971014	0.036363636	0.045977011	0.064516129	0.06779661	0.043478261	0.071428571	0.2
Manufacturing technology	0.068965517	0.052631579	0.057142857	0.5	1	0.028985507	0.018181818	0.022988506	0.032258065	0.033898305	0.02173913	0.035714286	0.1
Therapeutic alternative	2.379310345	1.815789474	1.971428571	17.25	34.5	1	0.627272727	0.793103448	1.112903226	1.169491525	0.75	1.232142857	3.45
Sci. evid. Clin.eff	3.793103448	2.894736842	3.142857143	27.5	55	1.594202899	1	1.264367816	1.774193548	1.86440678	1.195652174	1.964285714	5.5
Benefits from use of medicine (safety aspects)	3	2.289473684	2.485714286	21.75	43.5	1.260869565	0.790909091	1	1.403225806	1.474576271	0.945652174	1.553571429	4.35
Cost effectiveness analysis	2.137931034	1.631578947	1.771428571	15.5	31	0.898550725	0.563636364	0.712643678	1	1.050847458	0.673913043	1.107142857	3.1
Budget impact analysis	2.034482759	1.552631579	1.685714286	14.75	29.5	0.855072464	0.536363636	0.67816092	0.951612903	1	0.641304348	1.053571429	2.95
Therapy cost	3.172413793	2.421052632	2.628571429	23	46	1.333333333	0.836363636	1.057471264	1.483870968	1.559322034	1	1.642857143	4.6
HTA recommendations issued elsewhere	1.931034483	1.473684211	1.6	14	28	0.811594203	0.509090909	0.643678161	0.903225806	0.949152542	0.608695652	1	2.8
Rationalization analysis	0.689655172	0.526315789	0.571428571	5	10	0.289855072	0.181818182	0.229885057	0.322580645	0.338983051	0.217391304	0.357142857	1
SUM	22.86206897	17.44736842	18.94285714	165.75	331.5	9.608695652	6.027272727	7.620689655	10.69354839	11.23728814	7.206521739	11.83928571	33.15

*Sci. evid. clin.eff* Scientific evidence for clinical efficiency, *Adv.tech.* Advancement of technology

**Table 4:** AHP results- normalized matrix

MCDA criteria	Indication uniqueness	Disease rarity	Disease severity	Adv.tech.	Manufacturing technology	Therapeutic alternative	Sci. evid. Clin.eff	Benefits from use of medicine (safety aspects)	Cost effectiveness analysis	Budget impact analysis	Therapy cost	HTA recommendations issued elsewhere	Rationalization analysis	Weight
Indication uniqueness	0.024540349	0.014020913	0.014608234	0.014867258	0.014915032	0.015760303	0.017901509	0.020706096	0.023441153	0.026396161	0.031745601	0.03841396	0.042687007	2.31%
Disease rarity	0.058466036	0.033404057	0.019141824	0.019481235	0.019543835	0.020651432	0.023457149	0.027132126	0.030715994	0.034588072	0.041597684	0.050335534	0.055934698	3.34%
Disease severity	0.053850296	0.055939809	0.032055685	0.017943243	0.018000901	0.019021056	0.021605269	0.024990116	0.028291047	0.031857435	0.038313656	0.046361676	0.051518801	3.38%
Adv.tech.	0.00615432	0.006393121	0.006660922	0.003728466	0.002057246	0.002173835	0.002469174	0.002856013	0.003233263	0.00364085	0.004378704	0.005298477	0.005887863	0.42%
Manufacturing technology	0.00307716	0.003196561	0.003330461	0.003389515	0.001870223	0.001086917	0.001234587	0.001428007	0.001616631	0.001820425	0.002189352	0.002649239	0.002943931	0.23%
Therapeutic alternative	0.106162012	0.110281337	0.114900898	0.116938252	0.117314019	0.068179368	0.042593244	0.049266229	0.055773779	0.062804658	0.075532637	0.091398732	0.101565636	8.56%
Sci. evid. Clin.eff	0.169243788	0.175810828	0.183175345	0.1864233	0.187022349	0.197621357	0.12345868	0.078540365	0.088914719	0.100123368	0.120414348	0.145708124	0.161916232	14.76%
Benefits from use of medicine (safety aspects)	0.13385645	0.139050382	0.144875045	0.147443882	0.147917676	0.156300528	0.177535622	0.112942343	0.07032346	0.079188482	0.095236803	0.11524188	0.12806102	12.68%
Cost effectiveness analysis	0.095391953	0.099093376	0.103244285	0.105074951	0.105412597	0.111386583	0.126519639	0.146341176	0.091119217	0.056433171	0.067869905	0.082126397	0.091261876	9.86%
Budget impact analysis	0.090776214	0.094298535	0.098248594	0.099990679	0.100311987	0.10599691	0.120397721	0.139260151	0.157654949	0.09764097	0.064585878	0.078152539	0.086845979	10.26%
Therapy cost	0.14154935	0.147041783	0.153201197	0.155917669	0.156418692	0.165283317	0.187738819	0.217151422	0.245834836	0.276824938	0.183109422	0.121864976	0.135420848	17.60%
HTA recommendations issued elsewhere	0.086160474	0.089503694	0.093252903	0.094906407	0.095211378	0.100607237	0.114275803	0.132179126	0.149638596	0.168502136	0.202650744	0.13487033	0.082430082	11.88%
Rationalization analysis	0.030771598	0.031965605	0.033304608	0.033895145	0.034004063	0.035931156	0.040812787	0.047206831	0.053442356	0.060179334	0.072375266	0.087578136	0.053526027	4.73%
SUM	1	1	1	1	1	1	1	1	1	1	1	1	1	100.00%

*Sci. evid. clin.eff* Scientific evidence for clinical efficiency, *Adv.tech.* Advancement of technology

The sensitivity analysis indicated that six out of 13 criteria were deemed as equally impactful in the appraisal process (Table 2). The VIP minimum values and maximum regret of the remaining seven attributes did not pass the threshold test. The sensitivity results indicated that the criteria of “clinical evidence” could be considered as the key contributor to the decision making process of the HTA Appraisal Body. Its VIP maximum value was above the score for any other variable. In addition to that, the maximal regret of the “clinical evidence” equalled zero. Both VIP maximum values and maximum regret for safety aspects, costs of treatment, and availability of alternative therapies were set at 0.5. Consequently, they can be added to the list of the most important attributes of the recommendation process as well. With a VIP maximum value above 0.8 and a maximum regret of 0.512, the criteria of “recommendations from other jurisdictions” can be also listed as a potentially impactful contributor to the decision making process.

The criteria of the “the advancement of technology” and “manufacturing costs” had their VIP minimum values set to zero. Both were associated with the maximal regret. Hence, they have the highest opportunity costs when listed among the key contributors to the HTA recommendation process.

Taking the VIP results into consideration, there is a significant uncertainty with regards to the inclusion of “disease severity” and “disease rarity” among the decision-making criteria of the Polish HTA Appraisal Body as well. The same is true for “the results of rationalization analysis” and “indication uniqueness”. While the minimum VIP value for all four criteria was below 0.5, the maximal regret was above 0.5.

## Discussion

As the differences in access to OMPs across EU settings become more and more visible, there is a growing understanding that new approaches should be implemented to ensure a more transparent and fair allocation of funds across all individuals who suffer from rare diseases. Despite some international initiatives, new P&R pathways tailored specifically to meet peculiarities of the value assessment of OMPs are still really scarce. The currently available HTA guidelines are mainly limited to the assessment of clinical and cost effectiveness as well as safety. In the field of rare diseases, the importance of the consideration of non-economic criteria in the P&R decision-making is especially raised. Therefore, it was interesting to investigate what kind of value attributes are considered in the HTA process when there are no guidelines designed for the assessment of OMPs.

The study proved that both clinical evidence and economic considerations (CEA, BIA, the cost of therapy) played an important role in the assessment of OMPs in Poland. The results were consistent across different MCDA methods, and both the AHP weights and the SLAM rankings lead to similar conclusions.

The Polish Appraisal Body tended to rank the clinical evidence as the most important factor in the decision-making process. Interestingly, the notion of cost of therapy constituted the second largest contribution to the HTA recommendation process followed by the safety aspects. The importance of these two criteria, however, was similar, as could be seen in the comparison of AHP weights and the results of the sensitivity analysis. Nevertheless, it should be noted that there were statistically significant differences between positive and negative HTA recommendations in terms of the deliberation of safety aspects, which received more attention in the latter group.

According to both SLAM and AHP methods, the existence of a comparator was the fourth most impactful factor in the recommendation process at the Polish HTA agency. This was more important than disease rarity and severity. Interestingly, the results indicated that the availability of a therapeutic alternative was also valued more than economic evaluations. The weights of both CEA and BIA were set to 9%. The sensitivity analysis provided more insight into the importance of economic evaluation. The maximum VIP results for the CEA were set to one, which are only the second criteria with such a high value. if a lower regret is set with CEA versus BIA, then the former was more influential in the recommendation process than the latter.

Even though the cost of therapy played such a significant role in the HTA appraisal process, the results of the budget impact analysis (BIA) did not contribute that heavily towards the HTA outcome. BIA’s maximal regret set above 0.5 indicates that there is a significant opportunity cost for its inclusion in the list of recommendation making criteria.

Oddly enough, HTA recommendations issued by other HTA agencies also played an important role in the Appraisal Body’s judgment. Their weight was almost as high as the one established for the economic criteria. In addition, it should be mentioned that the influence of other HTA agencies was greater for the negative rather than the positive HTA recommendations of the Polish Appraisal Body in a statistically significant manner.

Although the study produced some interesting results, it is not free from limitations, some of which are inherited within the MCDA methodology itself. Firstly, the consequences of the adaptation of the SLAM bring certain simplifications to the analysis that are not without an impact on the results. The SLAM assumes mutual preference independence, which means that preference for an outcome measured by one criterion is independent from the outcome related to another criterion. It can be envisaged, however, that the real value judgment of the HTA Appraisal Body may not be independent across different criteria in the same manner in which value attributes correlate with each other. SLAM introduces a complete compensatory rule that involves an offset mechanism where a bad performance on one criterion can be compensated by a good performance on others. In real life settings, it is, however, difficult to expect that the HTA Appraisal Body will be willing to trade the value of a given OMP between two different attributes. Secondly, the adaptation of AHP methods was applied without transforming the scale to Saaty’s integer values 1–9. There are a number of approaches, which allow for this kind of switch with the adoption of the geometric scale [19], balanced scale [20], power [21], logarithmic [22], and finally root square [21]. It has to be underscored, however, that the analytic hierarchy process (AHP) method was selected following great consideration as it allows a pair-wise comparison of attributes. The application of AHP in this setting allowed the identification of intransitivity of collected criteria in the recommendation making process as well.

In addition to the limitations inherited in the MCDA methodology itself, there are also some additional risks of bias in our results related to the study design. Firstly, although we have taken into consideration all drug indication pairs assessed by the HTA agency between 2011 and 2015, we have not accounted for any potential changes in the Appraisal Body’s preferences across the study period. Secondly, two reviewers subjectively conducted the assessment of attitudes towards different MCDA criteria. Thirdly, the study was limited to the HTA recommendations only. Some of the Appraisal Body’s considerations failed to be presented in these documents, and the assessment of other P&R decision makers were left outside of the scope of our analysis.

Despite these limitations, our study contributes to the growing body of literature focused on the adaptation of MCDA to the OMPs assessment. In contrast to other publications, it presents the application of MCDA to the analysis of preferences of decision makers towards the value attributes of OMPs. To our knowledge, none of previous studies had a similar objective. For instance, Schey et al. [23] adopted MCDA framework to the value OMPs from the UK perspective. Iskrov [24] developed an MCDA value model for the assessment of OMPs from the perspective of the Bulgarian payer. In the manuscript of Sussex J [25], the objective was to pilot the use of MCDA as a framework for a value assessment of OMPs.

Still, the current study could be regarded as a continuation of earlier research that verified the potential impact of the implementation of an MCDA approach on the value assessment of OMPs in the Polish context [14]. Despite different objectives and the scope of data analysed, there is some resemblance in the conclusions between both studies. Firstly, it was revealed that the economic arguments had an immense importance in the Polish HTA process. Secondly, it was indicated that other criteria beyond the set of value attributes defined in HTA guidelines, such as the cost of therapy, HTA outcomes from other jurisdictions, played a role in the appraisal process also in Poland.

In addition to MCDA studies, there are some publications that provide insight into the value attributes used by manufacturers in price setting for OMPs as well. Therefore, some comparisons can be made between the preferences of decision makers and producers. In this regard, at least three interesting observations should be noted. Firstly, the opposite valuation was found with respect to the criteria of the clinical effectiveness. Although the highest importance of data regarding efficacy in the recommendation process was clearly expressed, it has not been found among the predictors of the price of orphan drugs [26, 27]. Secondly, some similarities could be drawn between the preferences of manufacturers and decision makers with respect to the availability of comparator. While it was valued as the fourth most relevant criteria in the HTA appraisal process, some studies indicated its importance in the manufacturers’ setting of a pricing policy. For instance, the Belgian comparative analysis of prices of reimbursed OMPs revealed that total annual costs were lower for drugs with a comparator compared to those without alternative treatment options [28]. Finally, a contradiction could be noted with respect to the notion of disease rarity. As this was not listed amongst the key attributes of the HTA appraisal process, the opposite could be said with respect to pricing studies. This review of 75 OMPs, which were granted marketing authorization by the EMA until 2014, revealed a significant inverse relationship between disease prevalence and the annual cost of drug treatment [29]. However, analysis of 45 OMPs in the five biggest EU jurisdictions revealed that the number of available alternatives as well as the prevalence of disease was correlated with the price of the drug [30].

In order to ensure the contribution of our findings towards further improvements of access to treatments for rare diseases, some recommendations for future development of value framework of OMPs can be elicited. The first recommendation is dedicated to further enhancement of the broader engagement of different stakeholders’ groups. Given the nature and peculiarities of rare diseases, uncertainty regarding both clinical and economic consequences of the implementation of a given health technology to the routine practice is inevitable. Therefore, it is not surprising that the Polish Appraisal Body attributed the highest value to the clinical evidence and noted the issue with credibility of data so many times among the reasons for negative HTA recommendation. To address such challenges, the development of a value framework should take views of both all decision makers and patient advocacy groups (PAGs) into consideration. With respect to the first group, the need of collaboration between the HTA agencies and EMA must be highlighted. Given the scarcity of clinical and safety data for OMPs, early dialog can be of great value especially when it comes to common understanding of unmet medical needs and scientific approaches to real life data collection. In addition to the wide range of incentives designed for OMPs available for the regular approval process, there are a number of special pathways for the marketing authorization such as accelerated, conditional, and exceptional pathways that enable the most needed OMPs to reach the patients in timely fashion [31]. Early engagement of HTA bodies in such processes could potentially facilitate a generation of P&R outcomes as well. As far as the contribution of the second group is concerned, it is again the lack of sufficient clinical and safety data that makes the PAG’s role in the P&R decision making so valuable. Given limited evidence regarding particular health states, patient experience can provide a needed insight into the real burden of disease. Such individual stories cannot compete with the breadth of evidence available for common diseases. Nevertheless, as long as it is ensured that patients’ stories are collected in a systematic and transparent manner, it can certainly successfully address some of the uncertainties of the HTA process. The second recommendation calls for the introduction of new innovative pricing & reimbursement arrangements. The results of our study indicated that the notion of the cost of therapy was a key consideration during the recommendation process of OMPs. At the same time, the price of a drug and an unfavorable cost effectiveness ratio were the most frequent reasons for the negative HTA recommendations. Therefore, a new set of P&R rules should enable the implementation of a special form of innovative managed entry agreements. Alternatively, a financing mechanism that links reimbursement with drug performance can be introduced to validate cost-effectiveness claims. Following the preferences of expert payers, the second one should especially be taken into consideration in the CEE Region [32]. Temporary access to OMPs could be secured as long as an HTA agency envisages the opportunity for further real life data collection before a final recommendation has been completed. For example, this is a common approach in the Netherlands and Sweden where temporary coverage is granted conditional upon the conduct of an observational study.

## Conclusions

In summary, it can be concluded that our study provides an interesting insight into the value judgment of the Polish HTA process. It also indicates that the decision-making preference of the HTA Appraisal Body goes beyond a standard set of HTA criteria. In addition to clinical and economic evidence, such criteria as the cost of therapy and the rationalization analysis, along with HTA recommendations from other HTA agencies, played a significant role without being included in the HTA guidelines. Therefore, there is a need for a new approach to the value assessment of OMPs that will even take into consideration those criteria that are not yet formally included in the HTA framework. Unless this happens, transparency and objectivity of the decision-making process cannot be fully ensured, and equity with regard to access to OMPs cannot be improved.

## Additional file

Additional file 1: MCDA analysis. (DOCX 9 kb)

## Abbreviations

AHP: Analytic hierarchy process
AHTAPoL: Polish health technology assessment agency
BIA: Budget impact analysis
CEE: Central and Eastern European
EMA: European medicines agency
EU: European Union
HTA: Health technology assessment
ISPOR: International Society for pharmacoeconomics and outcomes research
LSDP: Life saving drug program
MAVT: Multiple attribute value theory
MoCA: Mechanism for coordinated access
OMPs: Orphan medicinal products
OV: Overall value
P&R: Pricing and reimbursement
SLAM: Simple linear additive model
TVF: Transparent value framework
VIP: Variable interdependent parameters
