# Supplementary material for: Revealed preferences towards the appraisal of orphan drugs in Poland - multi criteria decision analysis
Source: Orphanet J Rare Dis. 2018 Apr 27;13:67. doi: 10.1186/s13023-018-0803-9 (PMC5922020; doi:10.1186/s13023-018-0803-9)
Supplement: Supplementary file 1 — MCDA analysis. (DOCX 9 kb) [file 13023_2018_803_MOESM1_ESM.docx]

*MCDA analysis*

*Weighted Summation*

A simple linear additive model (SLAM) was applied. This produces a unique value V(a) that indicates the strength of a given alternative taking into account all attributes. It is constructed by the aggregation formula as the sum of all partial values, each of which describes the performance of a given alternative against each attribute separately. The transition from partial to global value functions implies the use of the inter-criterion weights, which represent the user’s valuation of the importance of each criteria. [33]. In this particular study, the following additive aggregation formula was used [34]:

(1)

Where;

V(a) –overall value (OV)

ω_i_- relative importance (weight) of the ith attribute

v_i_ – the performance value score (partial value functions) of the ith attribute

For the purpose of this study, drug-indication pairs were treated as attributes and MCDA criteria as alternatives. As such, the MCDA matrix was constructed so that rows represented drug-indication pairs and columns represented MCDA criteria. Given the normalization constraint, all ω_i_ had to sum to 1. Therefore, in order to ensure the equal contribution of every HTA report, a weight for each drug-indication pair was set to (0.0179). Due to rounding, the last one in the alphabetic order, Xagrid CML, received a different weight (0.0155) [35]. As each MCDA criteria was assigned a unique value V(a), a ranking of MCDA criteria aligned with revealed preferences of HTA agency could be established.

*Analytic Hierarchy Process (AHP)*

In a similar fashion to SLAM, the Analytic Hierarchy Process develops a linear additive model. The frequency of utilization of MCDA criteria in the HTA recommendations was compared pairwise and a matrix of relative importance was established. The paired comparisons were organized into a positive reciprocal matrix.

The normalized matrix was formed by dividing the elements of each column by its sum i.e. T1, T1.Tn respectively. As a result the sum of each column was equal to 1 (Table 3).

A normalized matrix was used to measure the intensity of importance of each MCDA criteria in a pair-wise comparison. Instead of the adaptation of Saaty’s scale from 1 to 9, the values were kept untransformed. As a result, the range was adjusted to the number of HTA recommendations, which was from 1 to 55. In this respect we followed other researchers who argued that the choice of the scale "depends on the person and the decision problem" [36].

In the last step the weight for each MCDA criteria was calculated

(2)

Where;

Rn- the sum of each row

n- numbers of rows

*Sensitivity analysis*

In the sensitivity analysis, both the Variable Interdependent Parameters (VIP) and maximal regret methods were utilized. This allowed weights to vary within their natural boundaries from zero to one. We used VIP to produce two types of results. Firstly, the minimum and maximum value for each V(a), and secondly, VIP addressed the question of which V(a) dominated. For instance aj “dominated” ai when the maximum difference between ai and aj was negative or zero [37].

(3)

The maximal regret was calculated as the maximum difference between V(ai) and V(aj). This can be considered as the opportunity cost i.e. amount of loss due to making a wrong decision [38].

(4)

For the purpose of this particular study we assumed a threshold of 0.5 for both VIP minimum value and maximal regret. As such, a given MCDA criteria was considered to be an impactful factor in the recommendation process only if the VIP minimum value was above and maximal regret below the established threshold (threshold test).
